# Simulation and optimization of scrap wagon dismantling system based on Plant Simulation

**DOI:** 10.1186/s42492-023-00134-7

**Published:** 2023-04-24

**Authors:** Hai-Qing Chen, Yu-De Dong, Fei Hu, Ming-Ming Liu, Shi-Bao Zhang

**Affiliations:** 1 School of Mechanical Engineering, Hefei University of Technology, Hefei 230009, China; 2 China Railway Materials Group Resources Technology Co., Ltd., Hefei 230088, China

**Keywords:** Plant Simulation, Production optimization, Wagon dismantling, Genetic algorithm

## Abstract

Based on the existing plant layout and process flow, a simulation analysis was conducted using the Plant Simulation platform with the utilization efficiency of each station and production capacity of the dismantling system as indicators. A problem with long-term suspension in the disassembly process was determined. Based on the two optimization directions of increasing material transportation equipment and expanding the buffer capacity, a cost-oriented optimization model is established. A genetic algorithm and model simulation were used to solve the model. An optimization scheme that satisfies the production needs and has the lowest cost is proposed. The results show that the optimized dismantling system solves the suspended work problem at the dismantling station and a significant improvement in productivity and station utilization efficiency compared with the previous system.

## Introduction

Recently, as the number of railroad materials in end-of-life recovery and recycling has increased annually, a company has researched scrap wagon dismantling systems [1]. By combining simulations with actual production to identify bottlenecks in the dismantling line, the wasting resources can be avoided, and the entire research process can be advanced scientifically and effectively.

The production simulation method simulates the production process based on the actual running logic of the production line [2], which is easier to get the optimization scheme of the production line compared with the intuitive method based on trial and error experience [3]. At present, the simulation optimization of production line is widely applied in many fields such as automobile processing [4, 5] and mechanical production [6]. Fang [7] believes that the research focus of this method lies in the deep combination with optimization methods or tools, and scholars at home and abroad have conducted relevant researches on this. The combination of simulation method and bottleneck analyzer can better determine the bottleneck of the production line [8]. After the bottleneck problem is determined, the bottleneck station can be optimized and improved by the model timing method [9]. In the simulation process, hierarchical simulation experiments can be designed to compare the combination schemes and select the best combination [10]. After the combination of simulation and layout tool, the equipment can be arranged under space limitation, and then the optimal logistics route can be obtained [11]. This method is effective in the optimization of actual production system. Li et al. [12] combined simulation method with management operations research to conduct accurate and effective simulation evaluation of missile assembly production line. Yang et al. [13] determined the most appropriate buffer increment of tobacco sorting system through multi-level experiment. Bučková et al. [14] described the design of logistics system through simulation and proposes a material flow, which improved the efficiency of logistics. After establishing an automotive assembly rework evaluation model based on the rework characteristics of the automotive assembly production system, Li and Guo [15] conducted simulation verification through Plant Simulation, adjusted the rework scheme within a reasonable range, compared it, and obtained the optimal rework evaluation scheme. However, when the production environment is more complex, it requires several experiments to enumerate each optimization scheme and compare them individually, which affects the production, and the genetic algorithm is effective in solving the optimal solution. References [16–18] conducted a study based on the genetic algorithm and provided a scientific method for determining the optimal production sequence of each station of the production line and solving the problem of balancing the utilization efficiency of each station. Yang et al. [19] solved the mathematical model of workshop layout by genetic algorithm and optimized other production factors by Plant Simulation, reducing the logistics volume by 63.5% and increasing the throughput by 42.0%. Kyriklidis et al. [20] solved the problem of feedstock proportioning for marine biofuel blending using the genetic algorithm. Shehadeh et al. [21] proposed and validated a mathematical model for minimizing the time and cost of earthworks based on the genetic algorithm. This study introduces the genetic algorithm to study the bottleneck optimization problem of a production system.

This study simulates the specific operation of a scrap wagon dismantling system based on the Plant Simulation platform, which combines experimental and actual production data based on the resource flow of the dismantling system, using the production capacity and station utilization efficiency as reference indicators, and identifying the blockage links of the dismantling system. Aiming at the optimization direction of the blocking link, the model is established with the production capacity as the constraint and the minimum optimization cost as the goal, and the optimal solution is obtained efficiently using the genetic algorithm. The data of each iteration are used as simulation parameters to obtain the production capacity and then determine whether the constraint conditions are satisfied. Compared with the previous listing of each optimization scheme, the number of experiments was greatly reduced, the objectivity of the final optimization scheme was increased, and the production efficiency was improved.

## Methods

### Modeling process

**Fig. 1:**
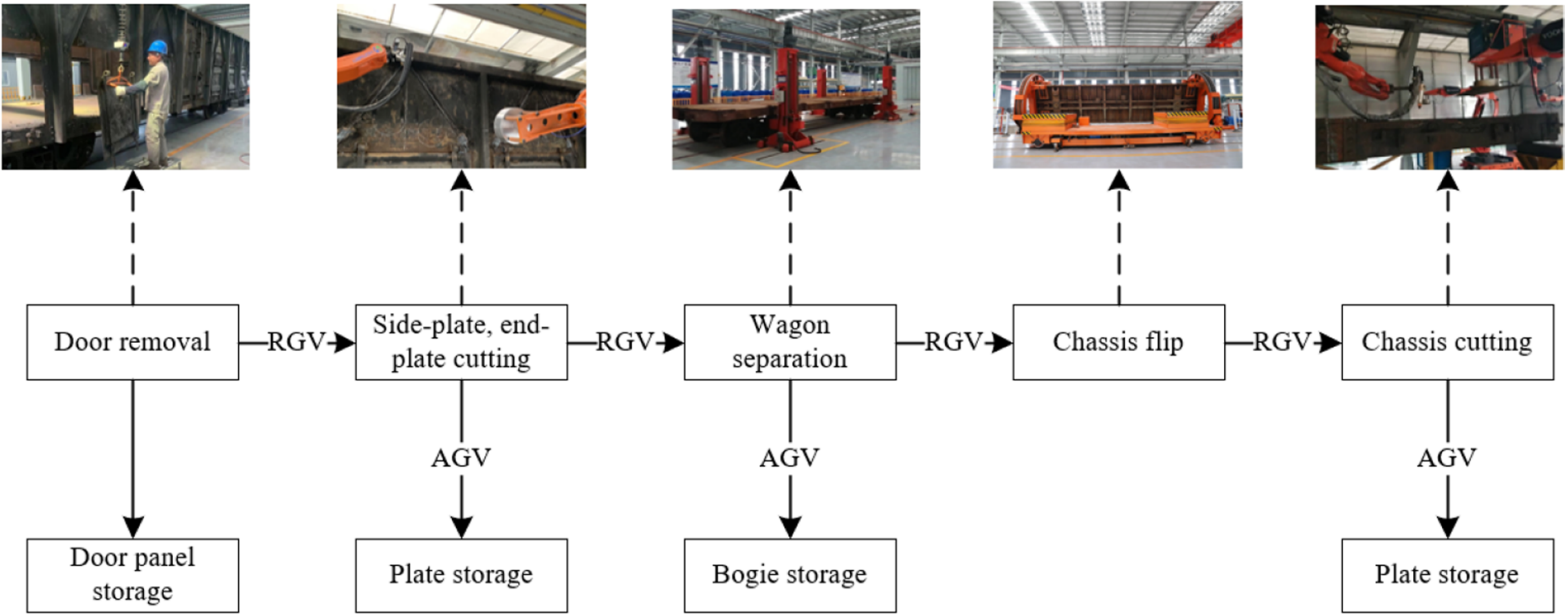
Wagon dismantling sequence diagram

The scrap wagon dismantling system is primarily divided into six parts: exterior cleaning, door removal, side-plate, end-plate cutting, wagon separation, chassis flip, and cutting. Figure 1 shows the dismantling process.

**Fig. 2:**
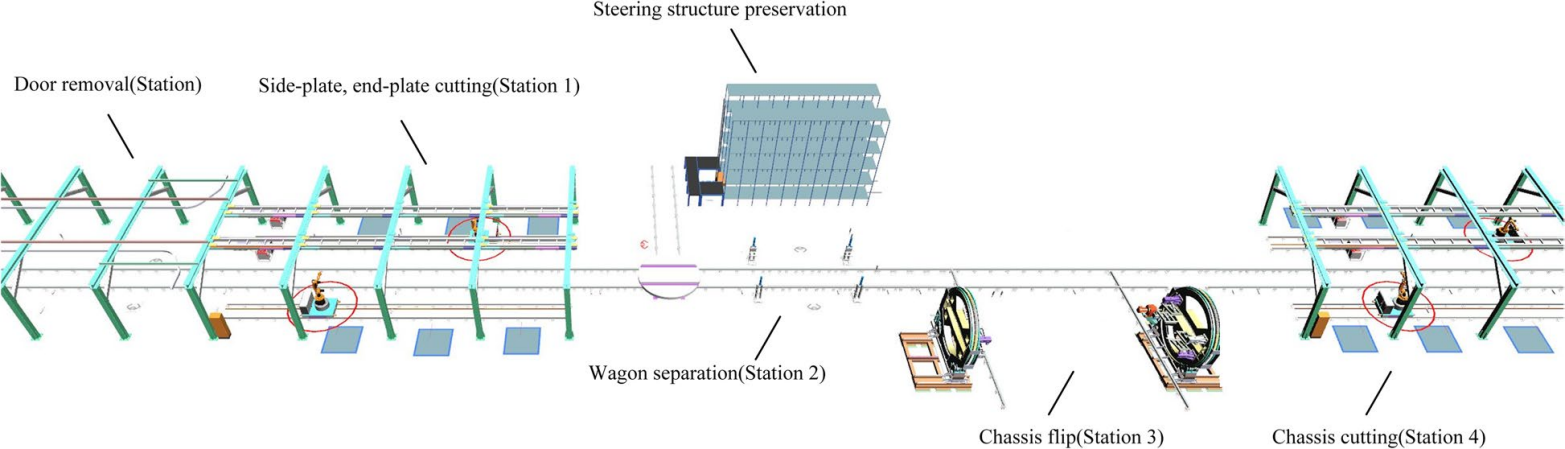
Wagon dismantling system 3D model diagram

**Fig. 3:**
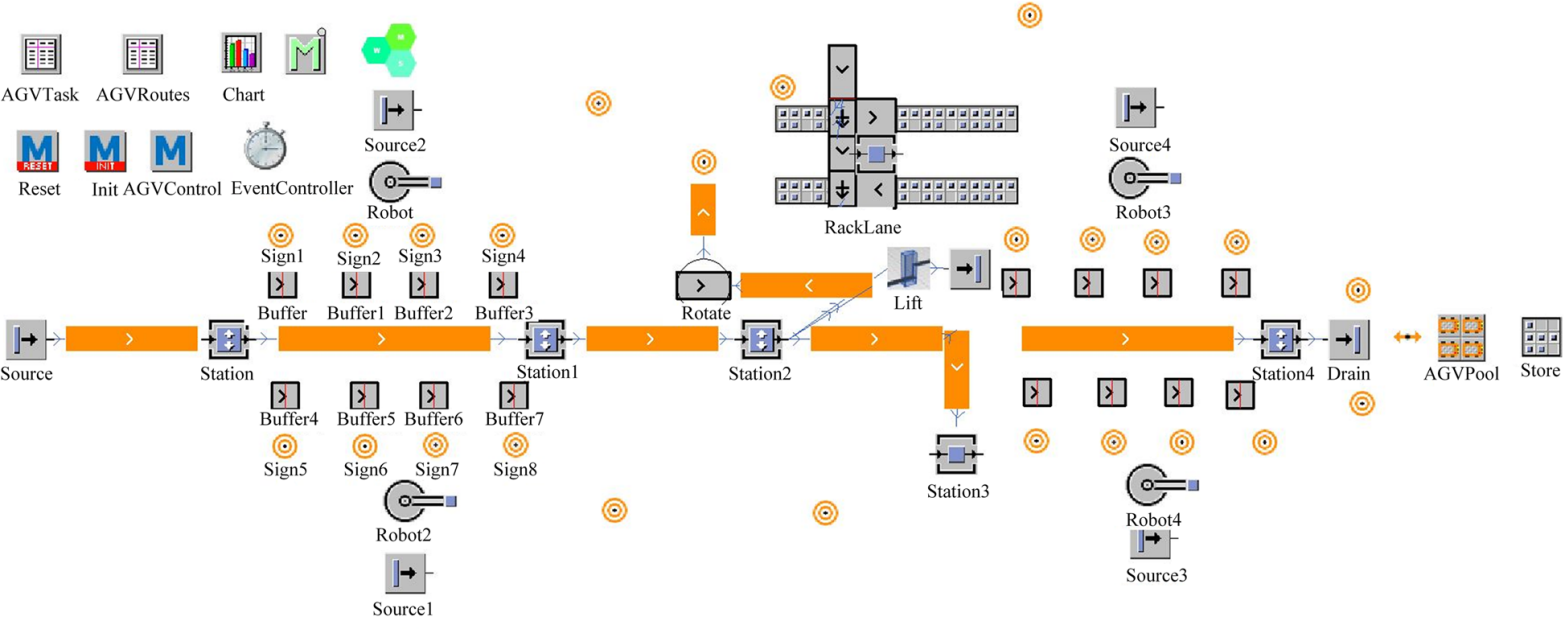
Wagon dismantling system 2D object diagram

The entire dismantling process is based on the rail. First, the discarded carriages are pushed into the disassembly line by the transport vehicle, the wagon doors are manually removed, and the carriage surface is cleaned. Then, the transport vehicle continues to push the carriage to the cutting station. According to the preset cutting path, the cutting robot cuts the side and end plates of wagon into fixed-sized by plasma cutting. Subsequently, the transport vehicle returns along the original road to transport the next carriage, and the rail guided vehicle (RGV) pulls the remaining carriage into the lift. After manually removing the steering structure, the remaining chassis is lifted to a certain height by the lifter and placed above the RGV, which is transported to the overturning mechanism by the RGV. The overturning mechanism turns the chassis by 180 degrees and continues to transport it to the chassis disassembly station for cutting. The steer and steering structures are carried into storage by automated guided vehicles (AGVs). Figure 2 shows the overall layout of the entire dismantling system, Fig. 3 shows the logical control view of the entire dismantling system, and Table 1 presents the functions of some of the modules in Fig. 3.

**Table 1:** Object menu

Object name	Function
Source	Scrap wagon
Source1、Source3	Material generated
RackLane	Save bogies
Sign	Paths
AGVTask	Material frame position
AGVRoutes	Record path marker points

According to the actual operation of the dismantling line, the Source object was set to generate a new wagon every 30 min. When the wagons to be dismantled enter Station1 and Station4 to cut them, Source1 and Source3 are activated. When the frame is full, the cutting station and material generation are suspended until the AGV updates the empty frame.

**Fig. 4:**
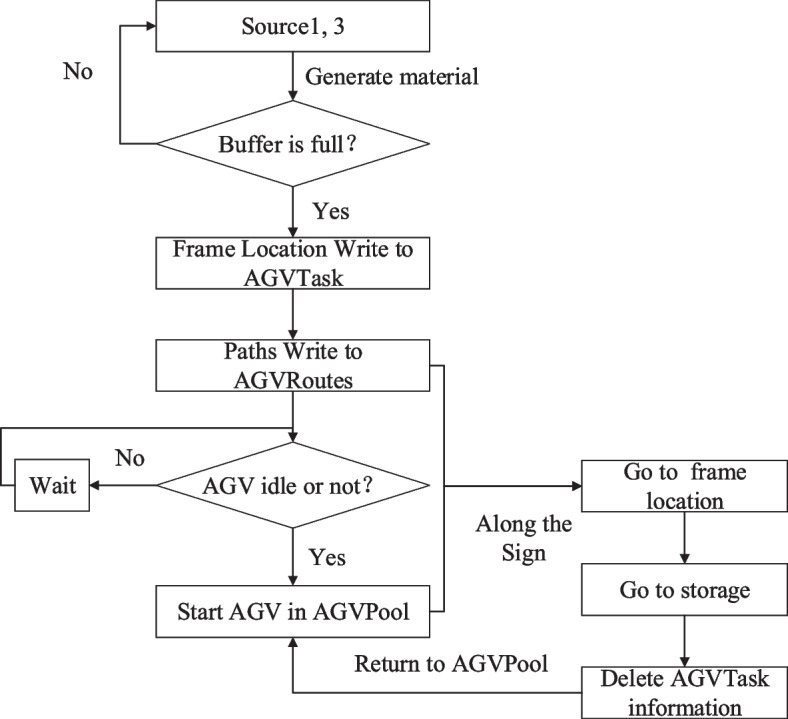
Control flow chart

Figure 4 shows the material flow control flowchart of the dismantling line. When the capacity of the frame reaches the upper limit, the current frame position information is written in the AGVTask. Path tags are read from AGVRoutes data is in AGVTask and an idle AGV is in AGVPool. After the transportation is over, the AGV status is changed to idle and the task information is erased in AGVTask.

The relevant parameters within the dismantling line are entered as shown in Table 2, setting the frame capacity within the dismantling process to 400 units and number of frames to 16.

**Table 2:** Simulation parameter input

Work process description	Process time (min)
Door removal	30
Side plate end plate cutting	112
Transfer between work processes	2.9
Wagon separation	11.4
Transfer between work processes	4.8
Chassis flip	14.4
Transfer between work processes	3.6
Chassis cutting	100
AGV loading and unloading time	0.1

### Bottleneck analysis

The yearly capacity of the disassembly line is determined to be 1234 carriages by simulation of the operation, and Table 3 shows the work rate of each work station at this time. The cutting proportion of Station1 and Station4 is far higher than that of the other stations, which is consistent with the actual production situation. Station1 and Station4 have an obvious pause in the production process, which significantly limits the production capacity of the entire disassembly system. By examining the disassembly system, it can be seen that Stations 1 and 4 are suspended because the material frame’s plates are constantly building up and the AGV is unable to transmit the frame with the full capacity to the logistics terminal in a timely manner. When all frames reach the capacity limit, the corresponding station is suspended, waiting for the AGV to update the empty frame and continue working.

**Table 3:** Work rate of each work station

Workstation name	Working (%)	Pausing (%)	Waiting (%)
Station	10.30	89.70	0.00
Station1	38.11	4.27	57.62
Station2	3.91	94.95	0.00
Station3	4.94	93.52	0.00
Station4	34.30	1.54	64.16

### Optimization methods

To address the production bottleneck of the dismantling process, the utilization rate of the process was improved by adding different types and quantities of equipment at different stations. The preliminary optimization ideas are as follows:

1. When the equipment type is 1, increase the number of this equipment in the simulation as 1, 2, 3, … and compare the production capacity to obtain the optimal result.
2. When the device type is *n* (*n* = 2,3,4,…), design the experiment and input parameters as {1,1,1,…,1},{1,1,1,…,2},… and analyze the experimental results.

However, this approach has certain limitations. In the parameter design, no stop condition is clearly established for the increase in the number of devices, and no clear evaluation standard is established for the optimal result, which can rely only on subjective judgment. When the optimization direction is greater and the gradient is small, a large number of optimization schemes are generated. If each optimization scheme is enumerated, the number of experiments will be excessive, which affects production. The genetic algorithm is widely used in production line balancing problems. By simulating the iteration of biological population genetic information, it can solve the problem of numerous experiments.

The analysis determined that the capacity of the dismantling line continues to increase with the number of single transport equipment and capacity of the storage equipment, and when the equipment is increased to a certain number, the capacity converges to a fixed value or the maximum capacity, M, of the dismantling line. Experiments were designed to obtain the critical values {*t*_1_, *t*_2_, *t*_3_…} for different devices, which will serve as boundary conditions for subsequent experiments. To quantify and analyze the results of the experiment, the increased annual cost C of the dismantling line system was introduced as an evaluation criterion, as shown in Eq. (1).

1 $$\text{C}=n_1C_1+n_2C_2+n_3C_3+\dots,$$

where C represents the increased cost of running the dismantling line for one year after the addition of equipment, n_*i*_ (*i* = 1,2,3,4…) represents the number of additions to each piece of equipment, and C_*i*_ (*i* = 1,2,3,4…) represents the annual cost of adding that equipment.

This study determines the optimal solution corresponding to {*n*_1_, *n*_2_, *n*_3_…} when the dismantling line reaches maximum capacity M, which is based on the concept of the genetic algorithm, and the cost C is taken as the minimum value. The specific steps are as follows.

1. Select binary coding as the coding method for chromosomes by sequentially splicing {*n*_1_, *n*_2_, *n*_3_…} into chromosome segments after coding them into one chromosome.
2. Initialize the population. The constraint is capacity M. The initial population is grouped equally according to the number of equipment species, *t*_i_ is encoded as the *i*-th chromosome segment of the i-th group of chromosomes, and the chromosome segments at the remaining positions are encoded following a random selection within the corresponding critical range: 0–*t*_*i*_, as shown in Fig. 5.

**Fig. 5:**
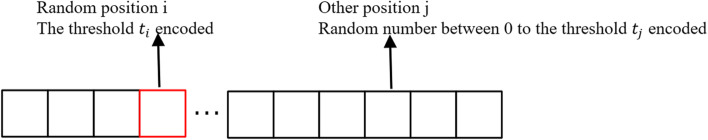
Schematic of chromosome initialization
3. Determine the fitness function. Because the objective of the iteration is the minimum value of C, the objective function is adjusted using the fitness function, as shown in Eq. (2),
  2 $$\text{f}=\frac{1}{C}$$
4. Determine the selection algorithm. Determine the algorithm to follow a conventional roulette-wheel rule. The probability of replicating each chromosome in the next generation is calculated using Eq. (3), and the length of *P*_*i*_ is assigned within [0,1], selecting *n* random numbers within [0,1] and determining the chromosome corresponding to the probability interval in which the random numbers are located to determine the next generation population.
  3 $${P}_{i}=\frac{{f}_{i}}{{\sum }_{i=1}^{N}{f}_{i}}(i=\text{1,2},3\dots n)$$
5. Perform chromosome fragment crossover swap. For the population to perform random two-by-two pairing, two integers, *i* and *j*, are randomly selected based on the crossover probability *P*_*c*_ in the chromosome length range, and the chromosome fragments of [*i*, *j*] of a pair of chromosomes are used as the offspring chromosomes after interchanging them.
6. Vary the chromosomal genes. To escape the local optimal solution and ensure the correctness of the final result, a mutation is performed with a considerably small probability *P*_*m*_ for the newly generated individuals. A mutated individual is obtained after the inversion of a randomly selected position on that chromosome.
7. Determine the termination condition. The termination condition is reached when the number of iterations reaches a specified number *a* or no change in the population, which is observed in *b* consecutive generations.

**Fig. 6:**
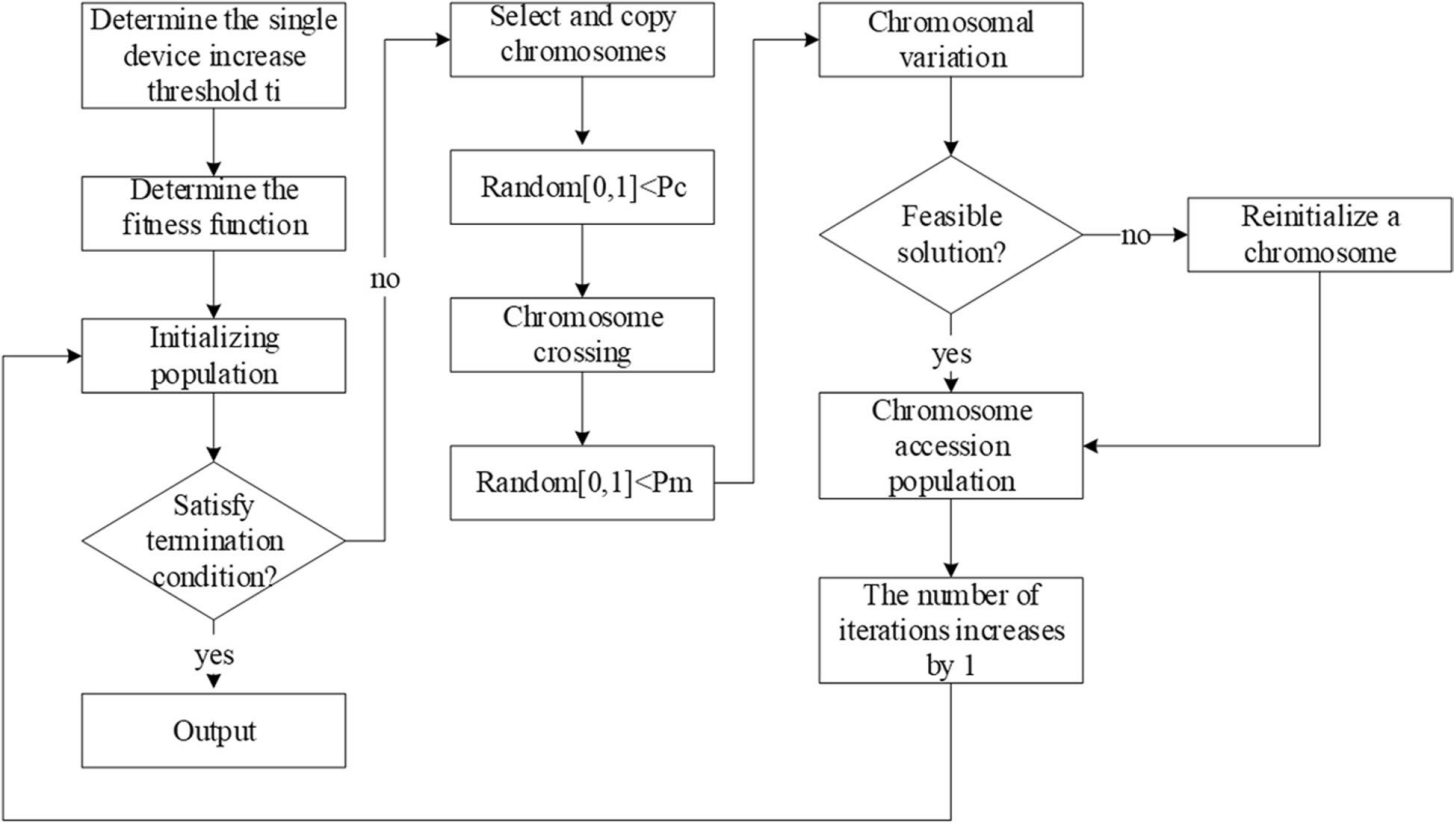
Optimization method flowchart

After the swap and mutation, the chromosome is decoded if the constraint is not satisfied, the chromosome is discarded, and a chromosome is reinitialized into the population. After several iterations, the optimal solutions are {*n*_1_, *n*_2_, *n*_3_…}. Figure 6 shows the flowchart.

## Results and Discussion

**Fig. 7:**
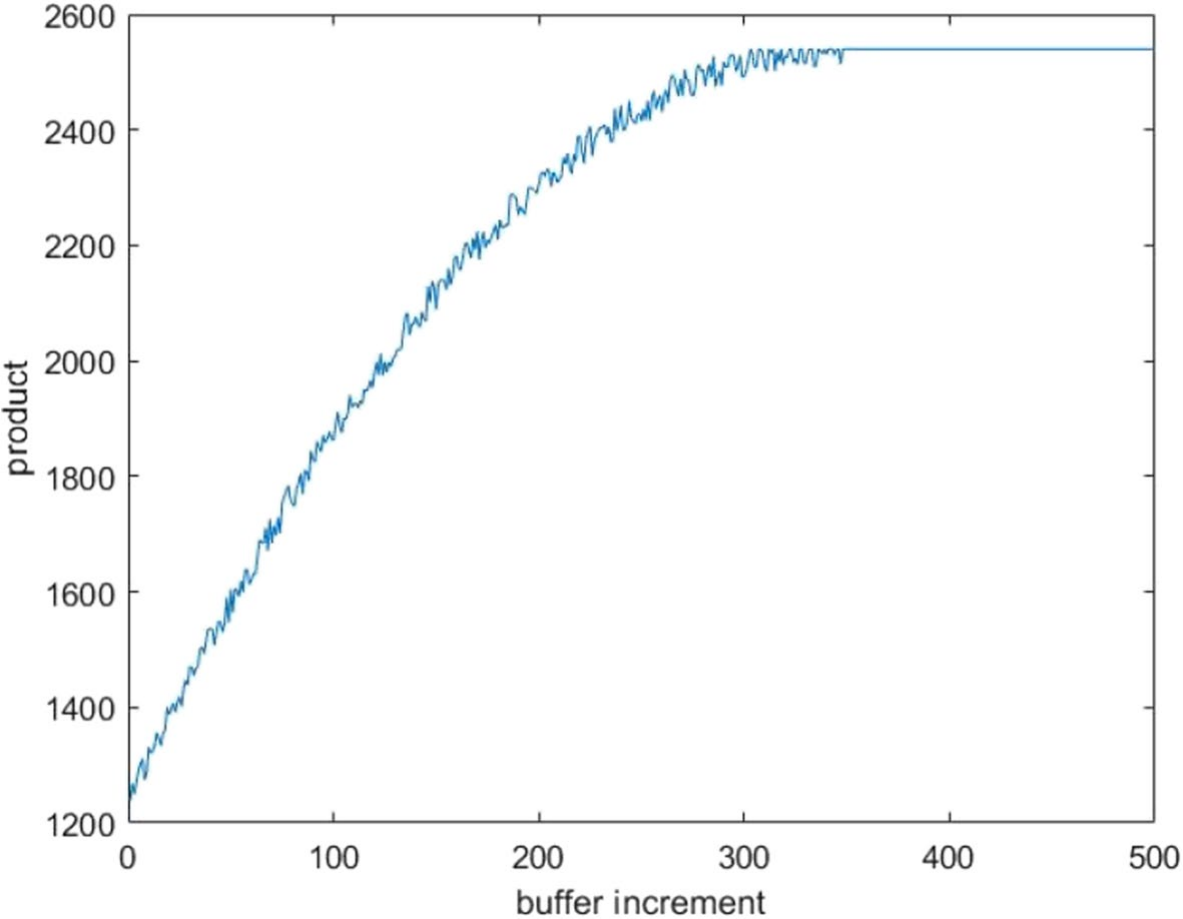
Variation of production with frame capacity

**Fig. 8:**
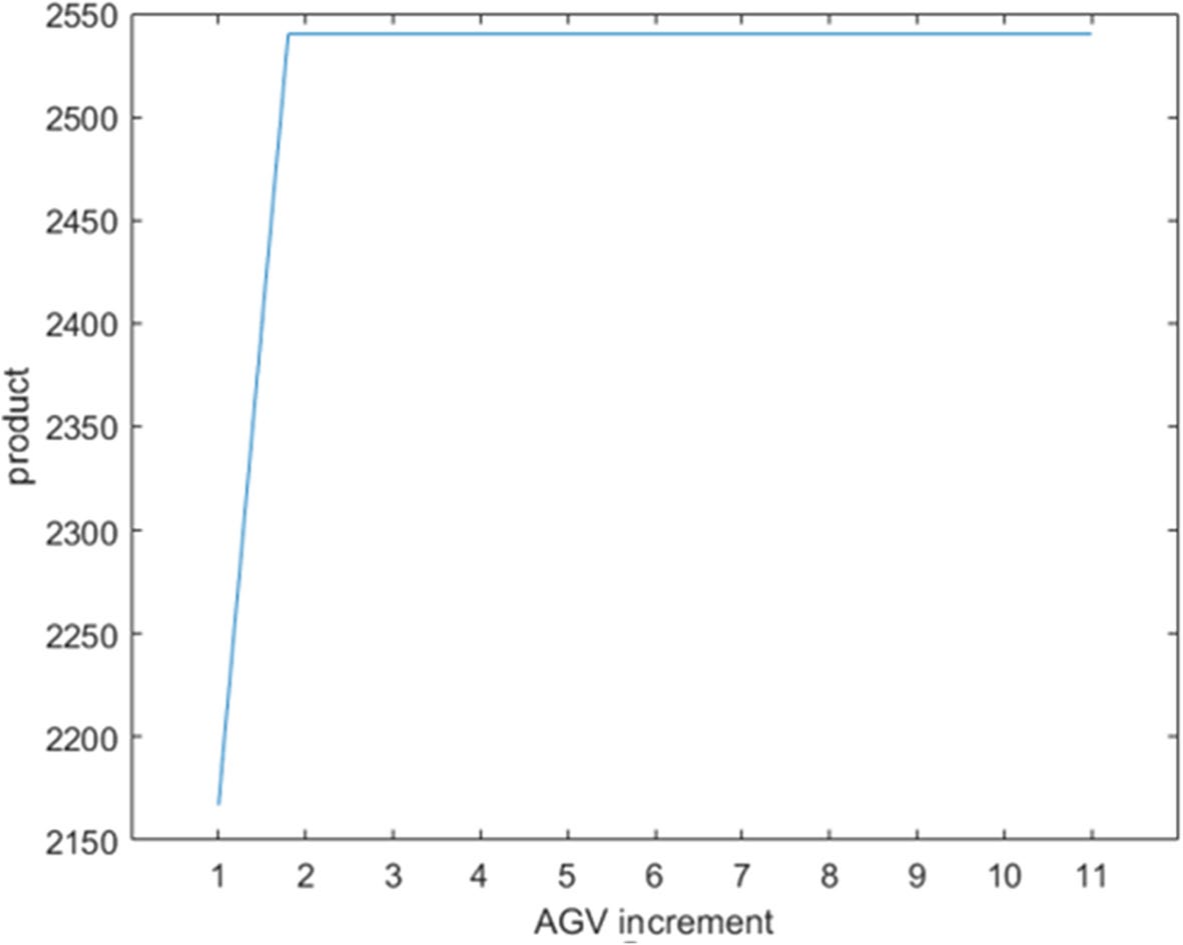
Variation of production with the number of AGVs

For the bottleneck problem of the dismantling line, two optimization directions can be followed, increasing the number of AGVs and capacity of the material frames. Through field research, the average annual cost C_1_ from adding one AGV is approximately 84000 RMB, and the average annual cost C_2_ from adding one unit of frame capacity is approximately 700 RMB. C_1_ includes the annual AGV purchase, site, and use and maintenance costs, and C_2_ includes the annual material frame purchase and site costs. As shown in Figs. 7 and 8, the critical value *t*_1_ for the increment of frame capacity is 347 units, the critical value *t*_2_ for the increment of AGVs is 2 units, and the maximum capacity M of the dismantling line is 2540 units after lifting the bottleneck. Equation (4) expresses the fitness function, where *n*_1_ is the increase in the number of AGVs, and n_2_ is the frame increase capacity.

4 $$\text{f}=\frac{1}{{84000n}_{1}+700{n}_{2}}$$

The initial population size was set to 30, crossover probability *P*_*c*_ was 0.6, variation probability *P*_*m*_ was 0.01, number of iterations was 300, and the iteration was stopped if* t* no change is observed in five consecutive generations. During the iteration, *n*_1_ and *n*_2_ are assigned to the number of AGVs (AGVPool.amount) and capacity of the frame (buffer. capacity), respectively, in the simulation model. After running the simulation, the simulation capacity (Source.stat) in the current solution is compared to the maximum capacity M to determine if the constraints were satisfied.

**Fig. 9:**
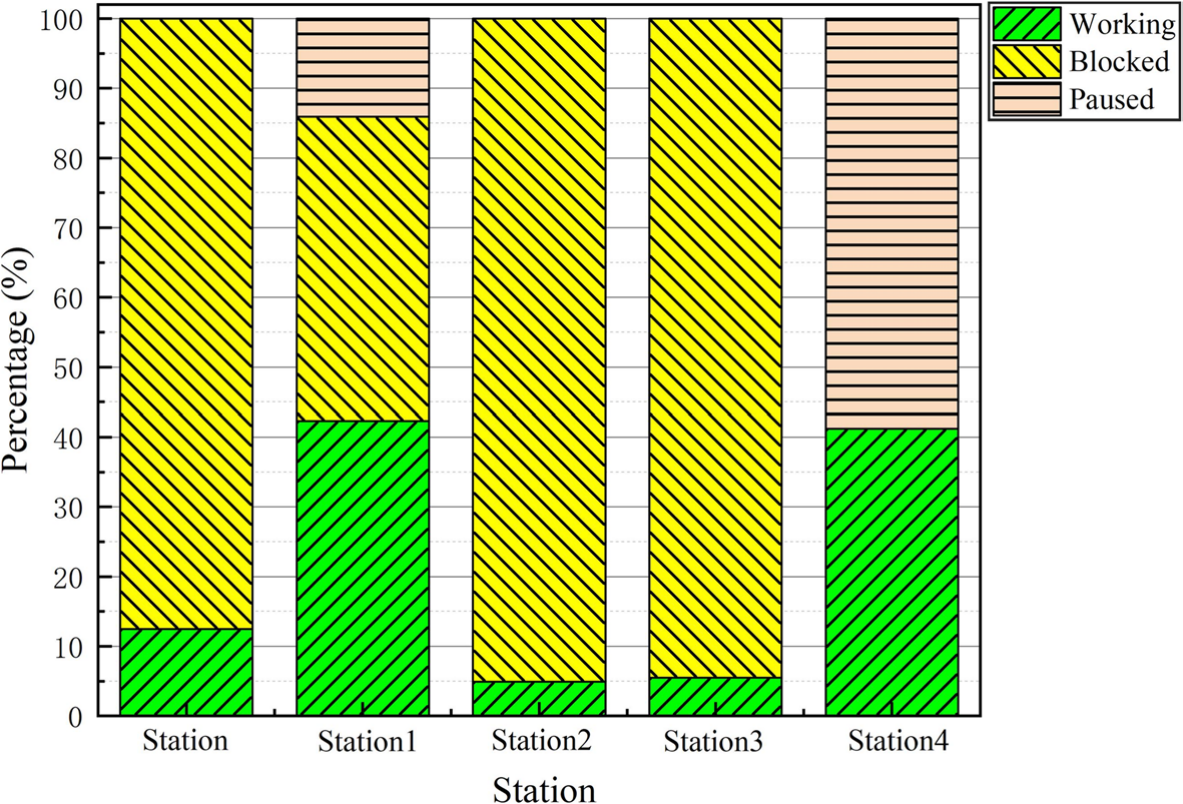
Work at workstation before optimization

**Fig. 10:**
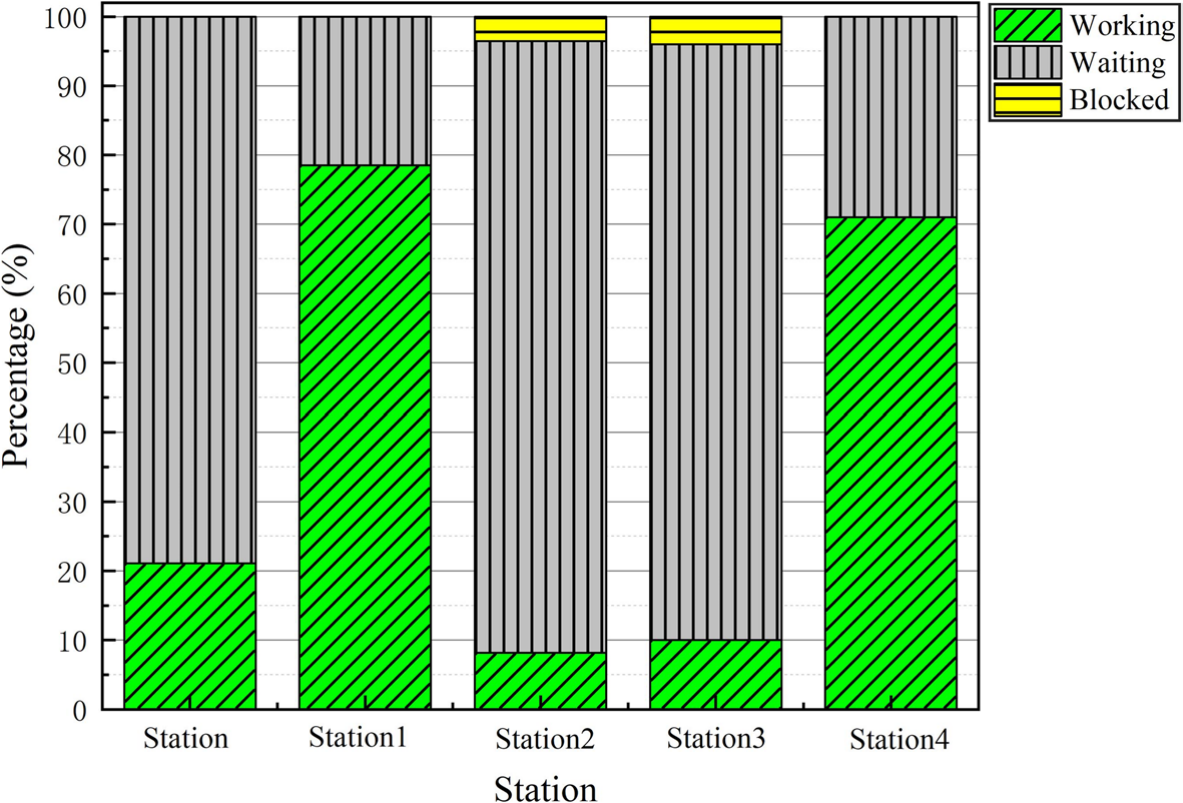
Work at workstation after optimization

Table 4 presents the simulation results of some dismantling line optimization schemes. The optimal solution for the dismantling line optimization is *n*_1_ = 1 and *n*_2_ = 67. That is, the increment in AGVs is 1 unit, the increment of material frame capacity is 67 units, and the increase in annual costs is C = 130900 RMB. When *n*_1_ or *n*_2_ is decreased by 1, the maximum production capacity cannot be reached, and when increased by 1, the annual costs cannot be lowered. The annual cost cannot be the lowest when only one optimization strategy is chosen to reach the maximum production capacity. Figures 9 and 10 compare the utilization rates of each station before and after optimization, respectively. The utilization rates of the Station1 and Station4 processes greatly improved, and the capacity of the entire dismantling line reached a maximum value of 2540.

**Table 4:** Comparison table of optimization options

Serial number	AGV increment (vehicle)	Frame capacity increment	Cost (RMB)	Production
1	1	67	130900	2540
2	1	66	130200	2411
3	0	67	46900	1929
4	2	67	214900	2540
5	1	68	131600	2540
6	2	0	168000	2540
7	0	347	242900	2540

The results verify the rationality of the proposed method and that it effectively solves the blocking problem of the scrap wagon dismantling system; however, limitations remain. First, the actual production environment is relatively complex, and omissions always exist when quantifying various optimization directions as costs. The production staff require careful communication, and possible cost changes introduced by the optimization plan must be considered as fully as possible. Second, during the establishment of the simulation model, some links that had a minimal influence on the results were ignored to simplify the model, and the actual simulation results may have a certain degree of deviation from the simulation results.

## Conclusions

This study used the Plant Simulation platform to model and assess a dismantling line based on the analysis of the operational process and the combination of data from the actual dismantling line. The analysis revealed that the bottleneck problem of the disassembly system is that the cutting station’s cutting surplus transportation capacity is insufficient, resulting in a long suspension of the cutting station. In response to this problem, combined with the actual production situation, two optimization directions are proposed: increasing the frame capacity and increasing the number of AGVs. With the goal of minimizing the optimization cost and the constraint of solving the problem of cutting station suspension, the model was established and the optimal solution was obtained using the genetic algorithm, that is, adding 1 AGV and 67 units of frame capacity. After optimization, the problem of cutting station suspension was solved, and the production capacity of the entire disassembly system increased by 51.4%.

## Data Availability

All data generated or analyzed during this study are included in this published article.

## Abbreviations

AGV: Automated guided vehicle
RGV: Rail guided vehicle
